# Indoor Visible Light Positioning System Based on Point Classification Using Artificial Intelligence Algorithms

**DOI:** 10.3390/s23115224

**Published:** 2023-05-31

**Authors:** Qianqian Long, Junyi Zhang, Lu Cao, Wenrui Wang

**Affiliations:** School of Electronic Engineering, Beijing University of Posts and Telecommunications, Beijing 100876, China; longqianqian@bupt.edu.cn (Q.L.); caolu_416@bupt.edu.cn (L.C.); wangwr@bupt.edu.cn (W.W.)

**Keywords:** visible light communication, indoor positioning, artificial intelligence algorithms, point classification

## Abstract

In RSSI-based indoor visible light positioning systems, when only RSSI is used for trilateral positioning, the receiver height needs to be known to calculate distance. Meanwhile, the positioning accuracy is greatly affected by multi-path effect interference, with the influence of the multi-path effect varying across different areas of the room. If only one single processing is used for positioning, the positioning error in the edge area will increase sharply. In order to address these problems, this paper proposes a new positioning scheme, which uses artificial intelligence algorithms for point classification. Firstly, height estimation is performed according to the received power data structure from different LEDs, which effectively extends the traditional RSSI trilateral positioning from 2D to 3D. The location points in the room are then divided into three categories: ordinary points, edge points and blind points, and corresponding models are used to process different types of points, respectively, to reduce the influence of the multi-path effect. Next, processed received power data are used in the trilateral positioning method for calculating the location point coordinates, and to reduce the room edge corner positioning error, so as to reduce the indoor average positioning error. Finally, a complete system is built in an experimental simulation to verify the effectiveness of the proposed schemes, which are shown to achieve centimeter-level positioning accuracy.

## 1. Introduction

### 1.1. Background

With the rapid development of mobile Internet and the global popularity of portable intelligent devices, the demand for accurate location information is increasing. Based on this, location-based services have consequently emerged, and play a vital role in daily human life [1]. At present, with the services of GPA (America) [2], Beidou Navigation System (China), GLONASS (Russia), Galileo Navigation System (Europe), and other satellite positioning systems, positioning accuracy can be at the meter-level which can meet the demand of daily positioning and location services in outdoor environments. However, in an indoor environment, satellite signals are greatly weakened and positioning errors are large so that it cannot meet the requirements of accurate positioning within smaller orders of magnitude indoors [3]. In order to make up for this defect in satellite positioning, indoor-positioning technologies proposed at present include: WLAN, Zigbee, Bluetooth, UWB, ultrasonic, RFID [4,5,6,7,8,9], etc. However, due to the increasingly tight spectrum resources available, the impact of electromagnetic interference, strong penetration which cannot guarantee security and other shortcomings in the field of traditional wireless communication, as a result, another visible light-positioning technology based on visible light communication has been proposed, bringing with it the advantages of high precision, low power consumption, anti-electromagnetic interference, high security, simple deployment (based on LEDs) and the provision of a large number of free spectrum resources [10].

Indoor visible light localization is mainly divided into two categories: one based on photo-detectors and the other based on image sensors [11,12]. Photo-detectors are more widely used owing to their low cost and low-power consumption. The existing indoor visible light positioning methods include RSSI, AOA, TOA, TDOA [13,14,15], etc. However, the angle-based and time-based positioning methods require additional synchronization equipment. RSSI only needs one single PD, hence lower hardware cost, which makes it the most widely used indoor visible light positioning method [16].

### 1.2. Motivation

However, in RSSI positioning methods, most researches based on traditional positioning methods are two-dimensional, and are difficult to extend to three-dimensional methods. This is because the distance calculation based on RSSI requires the known height difference between the transmitter and the receiver [17]. If the height is obtained by other sensors, hardware costs and system deployment difficulties increase. For example, an operable laser was used in [18] to conduct positioning calculation based on the angle between the laser and the receiver as well as the RSSI. In reference [19], a lifting rod was used to intentionally change the height of the receiver to implement extended 3D positioning.

In this case, a large number of artificial intelligence algorithms have been used in the researches of indoor visible RSSI localization methods. However, in these applications, the positioning process is too closed for human observation. For the genetic algorithm, particle swarm optimization algorithm, cuckoo algorithm, whale algorithm and other meta-heuristic algorithms [20,21,22,23], significant computational power of the receiving terminal is required, and a random number of iterations are needed for each positioning. Regarding another machine learning algorithm based on neural networks [24,25,26], it suffers from poor portability. If the indoor environment is changed, the network model needs to be re-generated, and if the positioning accuracy is to be improved, the network complexity will increase exponentially.

In addition, although studies have been conducted considering indoor visible light multi-path effects [27,28], these studies employed one method to process all data of the whole room, and there were no different treatments according to the different multi-path effects in different areas of the room.

Therefore, to address the aforementioned issues, this paper proposes a novel indoor visible light positioning system based on point classification using artificial intelligence algorithms. In this novel positioning system, we design three successive procedures, including height estimation procedure to extend the traditional RSSI trilateral positioning from 2D to 3D, pointing classification procedure to divide indoor positions into different types and a received power mapping procedure to reduce multi-path effects, respectively, and artificial intelligence algorithms are used in each procedure.

### 1.3. Contributions

The main contributions of this research can be summarized as follows:

1. A novel VLP RSSI positioning system is proposed by dividing positioning points into different types, which can reduce the average positioning error of the room, and artificial intelligence algorithms are used in different procedures, which can greatly improve the observability of the positioning process and make it more optimizable and improvable. To the best of our knowledge, this is the first study to divide the positioning process into different procedures and use artificial intelligence algorithms in each procedure.

2. A height estimation scheme is proposed to effectively extend the traditional RSSI trilateral positioning from 2D to 3D, without incurring additional hardware costs. Unlike previous works that calculate the coordinates at the same time by using artificial intelligence algorithms, in this paper we investigate height estimation alone for the first time.

3. According to the multi-path effect distribution in the room, we propose a novel indoor positioning point type division scheme, which divides the points into ordinary points, edge points and blind points, and then reduce the multi-path effect, respectively, in a subsequent procedure receiving power mapping. To the best of our knowledge, this is the first study to consider the varying influence of multi-path effects in different areas of a room.

4. Finally, in order to provide valuable engineering insights into the VLP system, a complete system is built in a simulation experiment to verify the feasibility and effectiveness of the proposed schemes, which can achieve centimeter-level positioning accuracy by using traditional artificial intelligence algorithms such as SVM and ANN. It is worth noting that the artificial intelligence algorithms mentioned in this paper constitute only part of the alternative algorithm examples for this system scheme. To further improve the positioning accuracy of this system, the algorithm structure and parameters can be improved or other intelligent algorithms can be selected for replacement.

The remainder of this paper is organized as follows. Section 2 introduces the basic indoor visible light channel model, the influence of the multi-path effect, and the traditional RSSI trilateral positioning method. The system scheme proposed in this paper is detailed in Section 3, including the training process of height estimation, point classification and RSSI receiving power mapping, and the overall positioning process. Section 4 provides the system simulation parameter settings, the simulation performance including the accuracy of each process, and the final positioning error comparison and display. Finally, Section 5 provides concluding remarks and suggestions for future work.

## 2. VLP System Model

### 2.1. Channel Model

**Figure 1:**
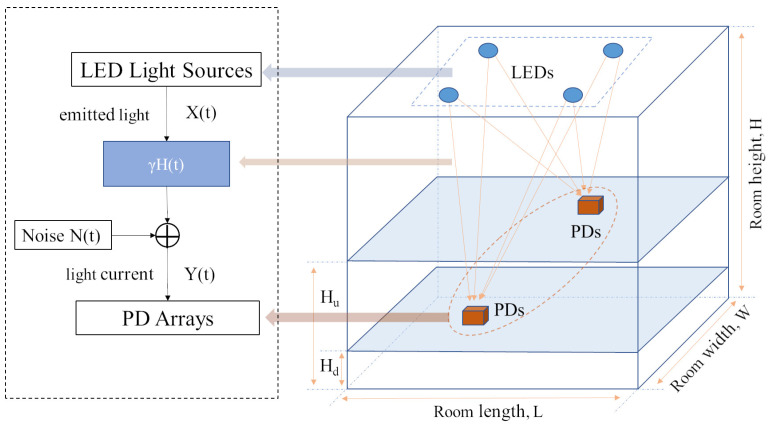
Overview of indoor visible light positioning system based on RSSI.

An RSSI-based indoor visible light positioning system mainly consists of LED light sources, visible light channels and PD arrays, as shown in Figure 1. The length, width and height of the interior space are L, W and H, respectively. In this paper, the dimensions of the room are set at 5 m × 5 m × 3 m. The reflection coefficients of the wall, ceiling and floor of the room are, respectively, set as 0.8, 0.8 and 0.3, and we define all the normal vector points to the inside of the room. Multiple LED array light sources are uniformly placed on the ceiling, and the receivers are located in the room space at a height between $H_{d}$ and $H_{u}$. According to [29], by conforming to common office settings, we set the receiver locations at heights ranging between 0.5 m and 1.5 m.

The indoor visible light channel model can be expressed as

(1) Y(t)=γH(t)·X(t)+N(t),

where Y(t) is the received signal, H(t) is the channel impulse response, γ is the photoelectric conversion efficiency of PD, X(t) represents the sent signal, and N(t) represents the noise.

**Figure 2:**
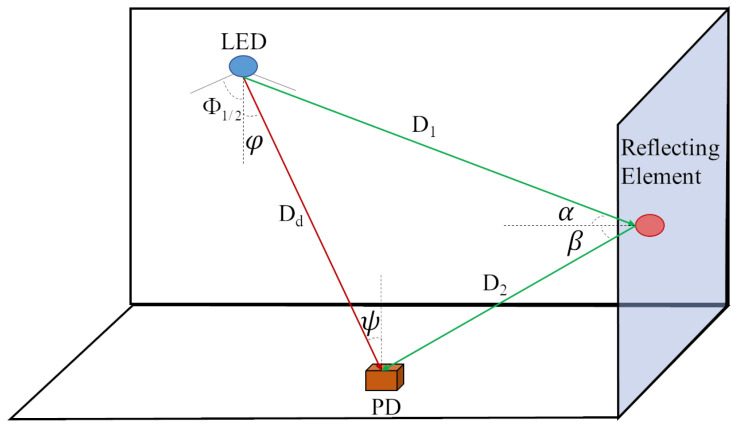
Overview of indoor visible light channel.

In the indoor visible light communication system, the communication links can be divided into direct link and reflected link, also called LOS link and NLOS link, as shown in Figure 2. For LOS links, the signal transmitted by the LED reaches the receiver directly without reflections or being blocked by obstacles. Channel gain in LOS link can be expressed as [30]:

(2) $$H_{LOS}\left(0;\;S,\;R\right)=\left\{\begin{array}{cc} \frac{(m+1)A}{2\pi D_{d}^{2}}\cos^{m}\left(\varphi \right)\cos\left(\psi \right)T_{s}\left(\psi \right)g\left(\psi \right) & 0\leq \psi \leq \Psi_{c}\\ 0 & \psi >\Psi_{c} \end{array}\right.,$$

and

(3) $$m=-\ln2/\ln(\cos\Phi_{1/2}),$$

where *S* and *R* represent the light source and PD, respectively. The Lambert scattering coefficient *m* is determined by the half power intensity angle $\Phi_{1/2}$ of LED. *A* is the area of PD, $D_{d}$ is the distance from the LED to the PD. φ and ψ are the divergence angle and the absorption angle, respectively. $\Psi_{c}$ presents the maximum field of view angle of PD. $T_{s}\left(\psi \right)$ and g(ψ) represent the gains of the optical filter and the condenser, respectively.

In the positioning system based on RSSI, the positioning accuracy is mainly determined by $P_{r}$, which is easily affected by the reflection of walls, floors and ceilings [31], so NLOS link must be taken into account. In the reflection link, the LED signal does not directly reach the PD in the transmission process, but may encounter some reflected objects and reaches the PDving end after the direction of the signal changes. The reflections may be singular or multiple in quantity. For single reflection shown in Figure 2, $D_{1}$ is the distance between LED and reflecting element, $D_{2}$ is the distance between the reflecting element and PD, α is the absorption angle of reflecting element and β represents the emission angle of the reflecting element. The channel gain of the first order reflection is given by

(4) $$H{\left(0\right)}_{NLOS}=\left\{\begin{array}{c} \frac{(m+1)Ar}{2\pi D_{1}^{2}D_{2}^{2}}\rho \cos^{m}\left(\varphi \right)dA\cos\left(\alpha \right)\cos\left(\beta \right)\cos\left(\Psi \right)T_{s}\left(\Psi \right)g\left(\Psi \right),0\leq \Psi \leq FOV\\ 0,\Psi >FOV \end{array}\right.,$$

where ρ is the reflectance factor, dA is the area of the reflecting element.

### 2.2. RSSI under Multi-Path Effect

**Figure 3:**
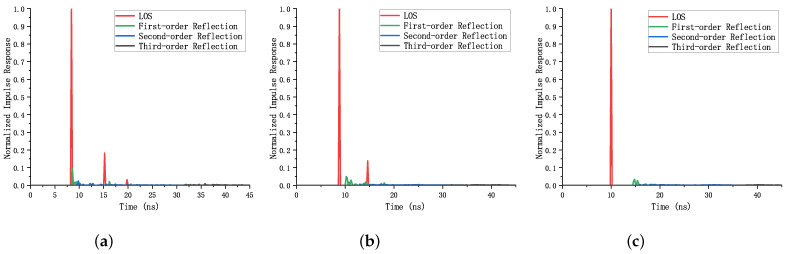
Normalized impulse response at different locations. (**a**) Normalized impulse response at (0.1, 0.1, 0.85). (**b**) Normalized impulse response at (0.5, 2.5, 0.85). (**c**) Normalized impulse response at (2.5, 2.5, 0.85).

In this paper, we simulate channel impulse response at different locations under four LED light sources (located at (1.0, 1.0, 3.0), (1.0, 4.0, 3.0), (4.0, 1.0, 3.0), (4.0, 4.0, 3.0), respectively), shown as Figure 3. Figure 3a shows the impulse response when the PD is located at (0.1, 0.1, 0.85), which represents the corner of the room; Figure 3b is the impulse response when the PD is located at (0.5, 2.5, 0.85), which represents the position near the edge of the room; Figure 3c represents the impulse response when the PD is located at (2.5, 2.5, 0.85), which represents the central area of the room.

Based on the above three results, it can be seen that the closer the PD to the room center is, the smaller the reflected component is. Conversely, the closer the PD to the corner of the room is, the larger the gain of the reflected component is. As can be seen from Figure 3a, even in the corner of the room with the greatest reflection influence, the first-order reflection component is only about 10% of the LOS component, the second-order reflection component is less than 2.5% of the LOS component, and the third-order reflection component is only about 0.4% of the LOS component. As can be seen from Figure 3b,c, the second-order and third-order reflection are both very small and almost impossible to be observed. Therefore, for simplicity of the system analysis, only first-order reflection is considered in this paper. The final total received power is the sum of the received power of LOS link and the received power of first-order reflection of NLOS link, which can be represented as

(5) $$P_{r}=\sum_{i=1}^{LEDs}\left\{P_{t}H_{LOS}\left(0;\;S,\;R\right)+\int_{walls}P_{t}H_{NLOS}\left(0;\;S,\;R\right)\right\}$$

### 2.3. Distance Calculation

In VLC channel, when the influence of multi-path effect is not taken into account, the receiving power is given by [32]

(6) $$P_{r}=\frac{(m+1)A}{2\pi D_{d}^{2}}\cos^{m}\left(\varphi \right)\cos\left(\psi \right)T_{s}\left(\psi \right)g\left(\psi \right)P_{t}$$

The distance between the LED and the PD can be represented as

(7) $$D_{d}=\sqrt{\frac{\left(m+1\right)A\cos^{m}\left(\varphi \right)\cos\left(\psi \right)T_{s}\left(\psi \right)g\left(\psi \right)P_{t}}{2\pi P_{r}}}$$

When the LED plane is parallel to the PD plane, the transmitting angle is equal to the receiving angle, which is

(8) $$\cos\varphi =\cos\psi =h/D_{d},$$

where *h* is the height difference between light source plane and receiver plane.

After substituting Equation (8) into Equation (7), the distance can be expressed as

(9) $$D_{d}=\sqrt[{m+3}]{\frac{\left(m+1\right)AT_{s}\left(\psi \right)g\left(\psi \right)P_{t}h^{\left(m+1\right)}}{2\pi P_{r}}}$$

### 2.4. Location Estimation Using Least Squares

Denote the coordinate of a receiver point as R(*x*, *y*, *z*), and the LED coordinates of the known transmitter are A($x_{1}$, $y_{1}$, $z_{1}$), B($x_{2}$, $y_{2}$, $z_{2}$), C($x_{3}$, $y_{3}$, $z_{3}$) and D($x_{4}$, $y_{4}$, $z_{4}$). During positioning, distance values $d_{1}$, $d_{2}$, $d_{3}$ and $d_{4}$ can be obtained. According to the trilateral positioning method, the following equations can be established [33]:

(10) $$\left\{\begin{array}{c} {\left(x_{1}-x\right)}^{2}+{\left(y_{1}-y\right)}^{2}+{\left(z_{1}-z\right)}^{2}=d_{1}^{2}\\ {\left(x_{2}-x\right)}^{2}+{\left(y_{2}-y\right)}^{2}+{\left(z_{2}-z\right)}^{2}=d_{2}^{2}\\ {\left(x_{3}-x\right)}^{2}+{\left(y_{3}-y\right)}^{2}+{\left(z_{3}-z\right)}^{2}=d_{3}^{2}\\ {\left(x_{4}-x\right)}^{2}+{\left(y_{4}-y\right)}^{2}+{\left(z_{4}-z\right)}^{2}=d_{4}^{2} \end{array}\right.$$

After mathematical transformation, it can be written in the following form

(11) Y=HX,

where

(12) $$Y=\left(\begin{array}{c} \left(d_{2}^{2}-k_{2}\right)-\left(d_{1}^{2}-k_{1}\right)\\ \left(d_{3}^{2}-k_{3}\right)-\left(d_{1}^{2}-k_{1}\right)\\ \left(d_{4}^{2}-k_{4}\right)-\left(d_{1}^{2}-k_{1}\right) \end{array}\right),X=\left(\begin{array}{c} x\\ y\\ z \end{array}\right)$$

(13) $$H=\left(\begin{array}{c} 2\left(x_{2}-x_{1}\right)\;\;\;\;\;\;\;\;\;2\left(y_{2}-y_{1}\right)\;\;\;\;\;\;\;\;2\left(z_{2}-z_{1}\right)\\ 2\left(x_{3}-x_{1}\right)\;\;\;\;\;\;\;\;2\left(y_{3}-y_{1}\right)\;\;\;\;\;\;\;\;2\left(z_{3}-z_{1}\right)\\ 2\left(x_{4}-x_{1}\right)\;\;\;\;\;\;\;\;2\left(y_{4}-y_{1}\right)\;\;\;\;\;\;\;\;2\left(z_{4}-z_{1}\right) \end{array}\right),$$

where

(14) $$k_{i}={x_{i}}^{2}+{y_{i}}^{2}+{z_{i}}^{2}$$

The least square method can be used to solve the approximate value of *X*, which is the solution of Equation (10)

(15) $$X={\left(H^{T}H\right)}^{-1}H^{T}Y$$

## 3. Proposed VLP Process

### 3.1. Height Estimation

**Figure 4:**
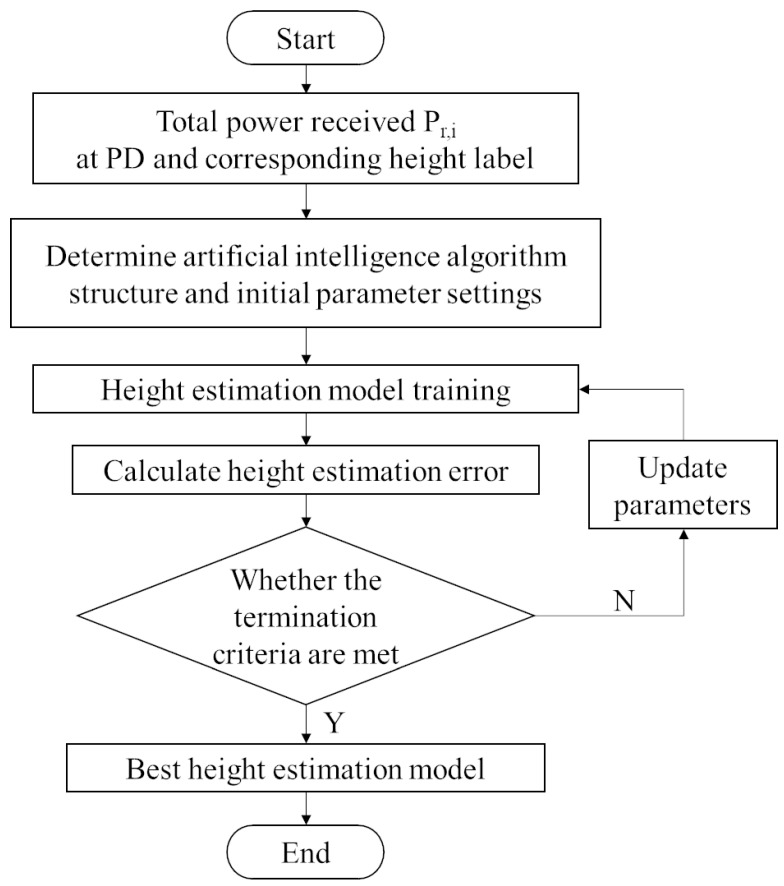
Flow of height estimation model training.

As can be seen from Equation (9), when only the received power intensity is obtained, the distance calculation requires the height difference between the LED light source plane and the PD plane to be known. However, in three-dimensional positioning, the height of the PD, namely the value of coordinate z, is unknown, which means the distance cannot be directly calculated by the Equation (9). Therefore, we considered using an artificial intelligence algorithm to explore the internal relationship between the PD height value and the RSSI from different LEDs received by the PD, and generated the height estimation model for the subsequent distance calculation process. Figure 4 shows the flow chart of training process of height estimation model proposed in this paper.

Firstly, the system model is built, the appropriate data composition is designed and selected, and the data set is generated. After the data set is normalized, it is put into the appropriate artificial intelligence algorithm for training. According to the test set results and errors, the height estimation model with the best performance is output for the overall positioning process.

The height range of the positioning area is $H\in [H_{d},\;H_{u}]$, dividing the height range evenly into $N_{H}$ parts according to the interval of $d_{H}$, that is $N_{H}=\frac{H_{u}-H_{d}}{d_{H}}$. The height plane set is $\left\{H_{1},H_{2},\ldots ,H_{N_{H}}\right\}$, where $H_{n}=H_{d}+n\times d_{H},n\in \left\{1,2,\ldots ,N_{H}\right\}$, and the corresponding height label set is $\left\{1,2,\ldots ,N_{H}\right\}$. Since the height is a continuous value, this paper considers using artificial intelligence algorithms such as regression algorithm.

### 3.2. Point Classification

In visible light communication, visible light signals are easily reflected by walls, floors and ceilings, leading to obvious multi-path effects in the room. The influence of multi-path effect differs across varying positions of the indoor area.

**Figure 5:**
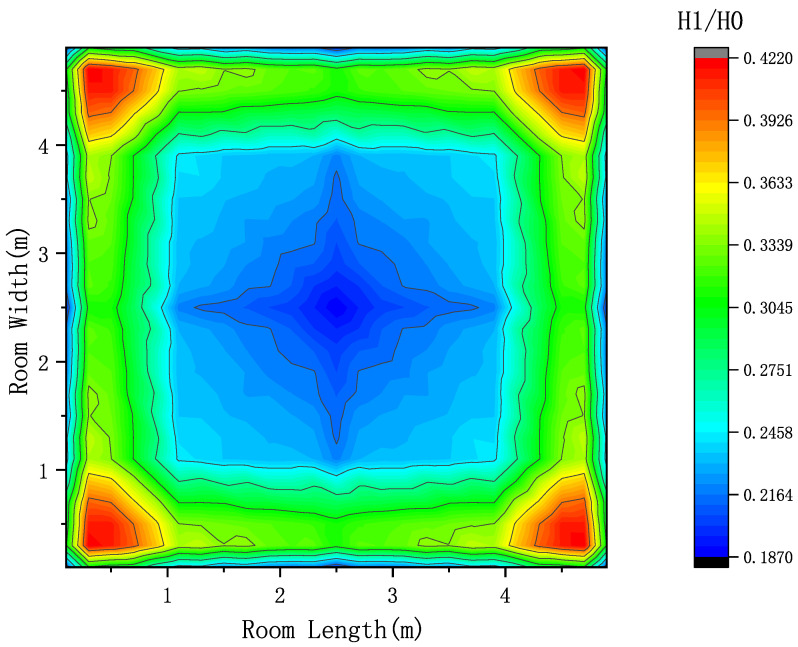
Ratio distribution of channel response between first-order reflection and LOS.

Figure 5 shows the channel response ratio distribution of first-order reflection to the LOS link on the indoor 0.85 m height plane. The colder the color of the color block, the lower the ratio, indicating that the first-order reflection at this position has less influence on the LOS link, that is, a weaker multi-path effect. On the contrary, if the color block is warmer, the ratio is higher, indicating that the first-order reflection at this position has a greater influence on the LOS link, which means the multi-path effect is stronger. It can be clearly seen from Figure 5 that the multi-path effect is strong at the four corners of the room and the area near the wall. The closer the area to the center of the room is, the weaker the multi-path effect is. Based on this, this paper considers that different positions in the room can be divided into ordinary points and edge points. At the same time, due to the hardware limitations of LED and PD, some areas of the room can not receive LOS signal from some LED light sources, so this part of the area is classified as a blind area. To sum up, this paper divides the indoor area into three categories: ordinary points, edge points and blind points, so that different data processing methods can be carried out for different types of position points in the next step to effectively reduce the multi-path effect.

**Figure 6:**
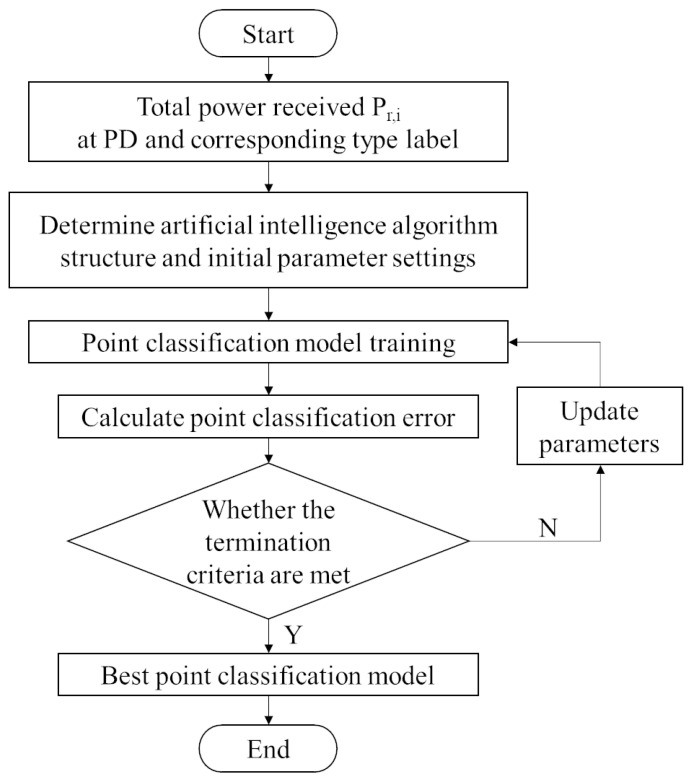
Flow of point classification model training.

The flow chart of training process of the point classification model proposed in this study is shown in Figure 6. Firstly, the system model is built, the appropriate data composition is designed and selected, and the data set is generated. After the data set is normalized, it is put into the appropriate artificial intelligence algorithm for training. According to the test set results and errors, the point classification model with the best performance is the output for the overall positioning process.

The specific criteria for point classification proposed in this paper are as follows:

The receiver coordinate is (x, y, z), the angles between the receiver and N LED light sources are $\left(\omega_{1},\omega_{2},\cdots ,\omega_{N}\right)$, then

(1) When the receiver is located near the wall, that is $x\in [0,d_{wall}]$ or $x\in [L-d_{wall},L]$ or $y\in [0,d_{wall}]$ or $y\in [W-d_{wall},W]$, or located at four corners of the room, that is $x\in \left[0,d_{corner}\right]\cap y\in \left[0,d_{corner}\right]$ or $x\in \left[0,d_{corner}\right]\cap y\in \left[W-d_{corner},W\right]$ or $x\in \left[L-d_{corner},L\right]\cap y\in \left[0,d_{corner}\right]$ or $x\in \left[L-d_{corner},L\right]\cap y\in \left[W-d_{corner},W\right]$, this receiver point is the edge point, and the label is set to 2;

(2) When $\exists \omega_{n}>FOV,n\in \left\{1,2,\cdots ,N\right\}$, the receiver is the blind point, and the label is set as 3;

(3) If the above criteria are not met, this receiver point is an ordinary point and the label is set as 1.

Among them, the blind point classification has the highest priority. In other words, if the receiver meet criteria (1) and (2) at the same time, this receiver point is categorized as a blind point and the label is set as 3.

Where $d_{wall}$ is the wall edge interval, $d_{corner}$ is the corner edge interval. Since the point type label values are discrete values, artificial intelligence algorithms such as classification algorithms are considered in this paper.

### 3.3. Received Power Mapping

**Figure 7:**
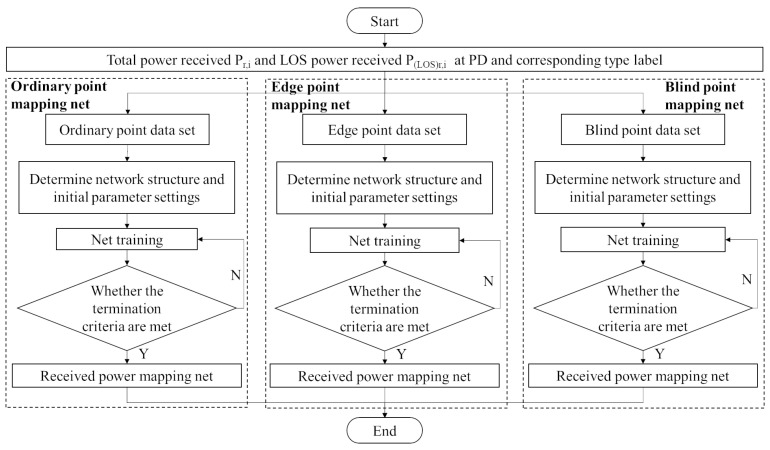
Flow of received power mapping net training.

The specific received power mapping training process is shown in Figure 7. According to the above different point classification label values, the room points are divided into different types, and the data processing to reduce the multi-path effect is carried out, respectively. In this paper, artificial intelligence algorithms such as artificial neural network are considered for processing, and the mapping network from the sum of received power containing first-order reflection to the received power of the LOS link is generated. In this paper, deep learning artificial intelligence algorithms such as artificial neural network and extreme learning machine are considered. In the process, the training parameters of the intelligent algorithm are determined by the size of the sub-data set and the value of the label. The training complexity of the following mapping models from high to low are: blind point mapping model, edge point mapping model and ordinary point mapping model.

### 3.4. Complete Process

**Figure 8:**
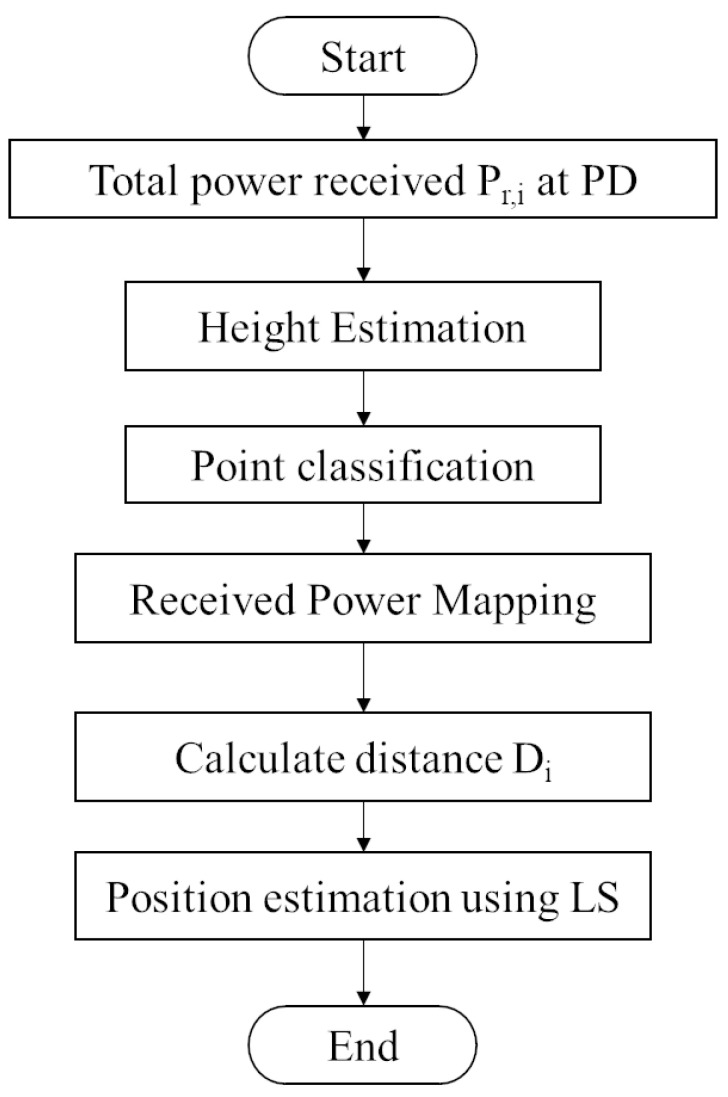
Flow of proposed VLP system.

A novel visible light positioning system based on point classification using artificial intelligence algorithms is proposed in this paper, and its overall process is shown in Figure 8. There are six steps involved as follows:

Step 1: Obtain the total received power data of LED light sources received by receiver points to be positioned. The total received power refers to the sum of the received power from the LOS link and NLOS link of one single LED light source received by one single receiver to be positioned.

Step 2: Put the total received power data into the height estimation model to obtain the height value of the points to be positioned.

Step 3: Put the total received power data into the point classification model to obtain the label value of points to be positioned.

Step 4: According to the labels of the points to be positioned obtained in step 3, put the total received power data of the points to be positioned into the corresponding mapping model to obtain the LOS link received power of the points to be positioned.

Step 5: Calculate the distances between the points and different light sources according to the height value obtained in step 2 and the LOS receiving power obtained in step 4.

Step 6: According to the distances obtained in step 5, the least square method is used to calculate the coordinates of points to be positioned.

Where height estimation model, point classification model and received power mapping model are obtained by the aforementioned processes.

## 4. Simulation Results and Discussions

The performance of the proposed positioning method is evaluated through computer simulations. The four LEDs are located on the ceiling of the room with a size of 5 m × 5 m × 3 m, and there coordinates are (1, 1, 3), (1, 4, 3), (4, 1, 3), (4, 4, 3), respectively. The receiver can be located at any place in the positioning area, whose coordinate is (*x*, *y*, *z*), where *x* ∈ [0, 5], *y* ∈ [0, 5], *z* ∈ [0.5, 1.5]. The simulation parameters are shown in Table 1, in which most parameters are the same as, or similar to those reported in [34,35,36].

The indoor visible light channel system is simulated to obtain RSSI data of 100 height planes and 625 points for each plane. Each group of RSSI data consists of the received power from 4 LEDs.

**Figure 9:**
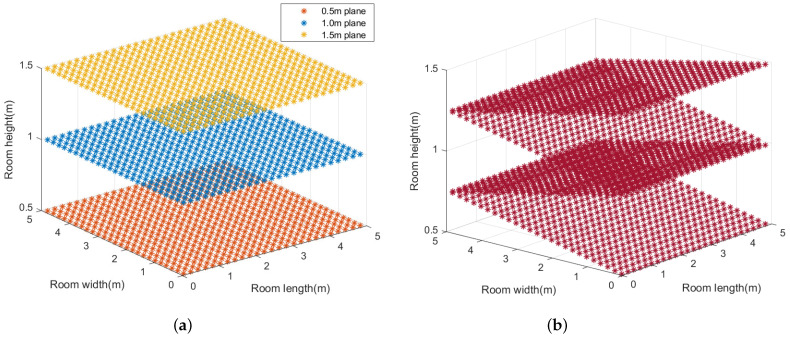
Data planes for simulation testing. (**a**) Data planes, respectively, at 0.5 m, 1.0 m, 1.5 m. (**b**) Zigzag plane.

In order to better evaluate the three-dimensional positioning effectiveness of the proposed visible light positioning system, there are four data planes selected in this study, as shown in Figure 9, including 0.5 m plane, 1.0 m plane, 1.5 m plane and zigzag plane.

### 4.1. Height Estimation Error

The data set used for the height estimation model training includes the input data, namely the RSSI data at the receiver from four LEDs, and the output data, namely the corresponding height label value. The artificial intelligence algorithms adopted in this study include linear regression, traditional SVR, PSO-SVR, GA-SVR and ANN, and the performance results are shown in Table 2.

When given sufficiently large range of c, g parameters for training, the traditional SVR can potentially achieve better results. While the training with large range of c, g would require significant computation resources, PSO and GA are introduced to optimize c, g values of SVR, which greatly shorten the training time and improve the accuracy of height estimation. If the ANN is designed properly, the training time and estimation accuracy can be enhanced.

**Figure 10:**
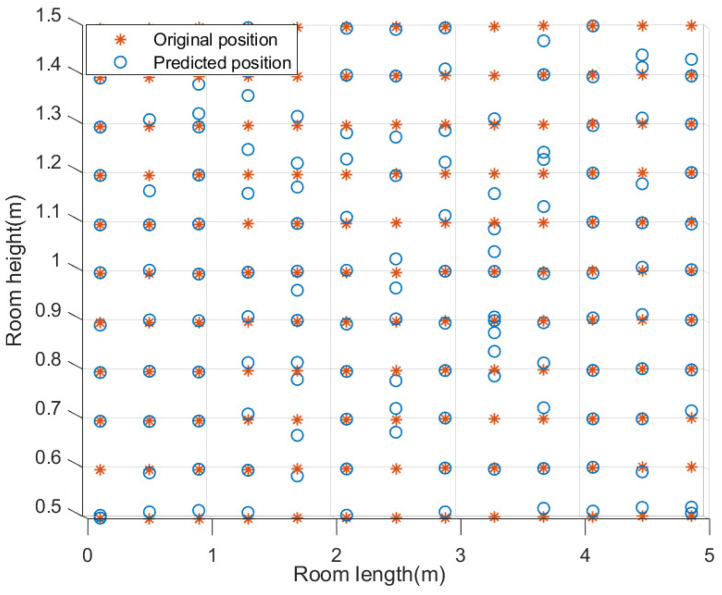
Performance of best height estimation model.

As a result, the height estimation model trained by PSO-SVR with the minimum height estimation error is selected in this paper for the subsequent positioning process. We take the height estimation of the room diagonal plane as an example to show the performance of the height estimation model, as shown in Figure 10. The minimum error is $1.6\times 10^{-3}$ cm, the maximum error is 34 cm, and the average error is 3.45 cm. It can be seen that those with large errors are distributed in the center of the room, that is because the center of the room is less affected by the multi-path effect, which means the received power from LED at similar heights is also similar, so that different height planes are difficult to distinguish.

The height estimation error of the four test planes is shown in Table 3. It can be seen that the height estimation error of the 1 m plane is the smallest, less than 1 cm, while the height estimation error of the 0.5 m and 1.5 m plane is larger. In general, the average height error of the positioning area in the room can be less than 2 cm.

### 4.2. Point Classification Error

The artificial intelligence algorithm adopted in this study is PSO-SVM, the specific parameter settings are shown in Table 4.

The data set used for the point classification model training includes the input data, namely the RSSI data at the receiver from four LEDs, and the output data, namely the corresponding height label value.

**Figure 11:**
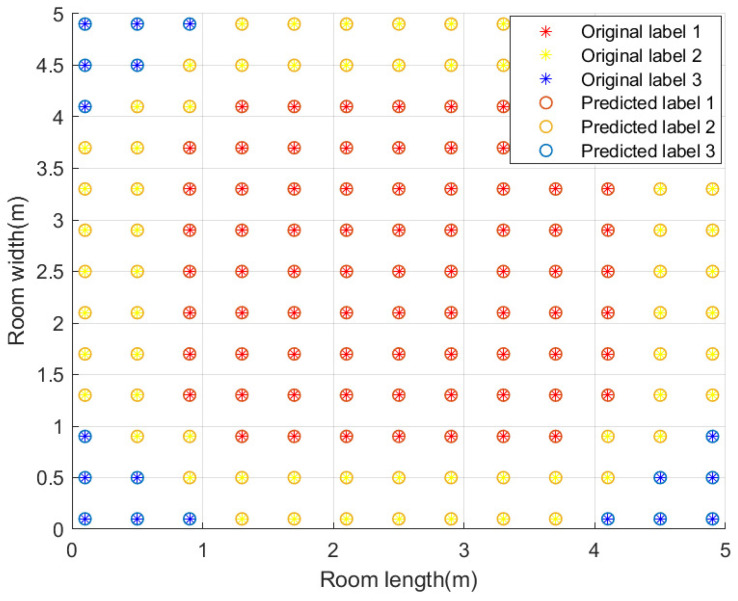
Performance of point classification model.

The overall point classification accuracy of the trained point classification model can reach 99.85%. We take the 1.2 m plane as an example to show the performance of the point classification model, as shown in Figure 11. It can be seen that the classification accuracy of the three point types is 100%.

The classification accuracy of the four test planes is shown in Table 5. It can be seen that the classification effect of 0.5 m and 1.5 m plane is slightly worse, reaching 99.52%, while the classification accuracy of 1m plane can reach 100%. The result of zigzag plane with multi-height is between the two, reaching 99.92%.

### 4.3. Received Power Mapping Error

The data set used for received power mapping model training includes the input data, namely the received power data at the receiver from four LEDs, and the output data, namely the corresponding LOS received power data. The artificial intelligence algorithm adopted in this study is artificial neural network. According to the point classification label value, the received power mapping data set is divided into three sub-data sets, namely, the ordinary point received power mapping data set, the edge point received power mapping data set and the blind point received power mapping data set, which are, respectively, sent into three neural networks with different parameter settings for training, and then compared with the mapping network trained with all data. The specific network settings and results are shown in Table 6.

As can be seen from Table 6, under the same network parameter settings, the training results of unclassified data are not as good as the training results of classified data. This is because the received power data obtained in the same type of positioning area are more similar, so the network training convergence is faster and the effect is better. However, the received power data in different areas are significantly different. Therefore, the result of training all data is less effective. Compared with one network training for all data, this scheme can reduce the network complexity and reduce the average error.

The mapping errors of the classified received power mapping network at the four test planes are shown in Table 7. It can be seen that the average mapping errors of the received power in all planes are kept below 4%.

Taking point (1.3, 2.1, 1) as an example, the relationship between the received power error and the positioning error is shown in Table 8. It can be seen that even when the error of the received power is 5%, the positioning error is only 3.27 cm. Therefore, without considering the error of height estimation and point classification, the performance of the above received power mapping network can meet the requirement of centimeter-level positioning.

### 4.4. Positioning Error through Complete Process

**Figure 12:**
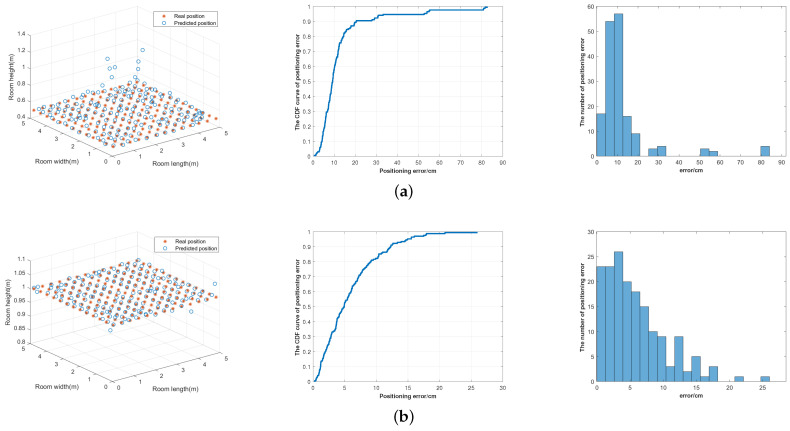

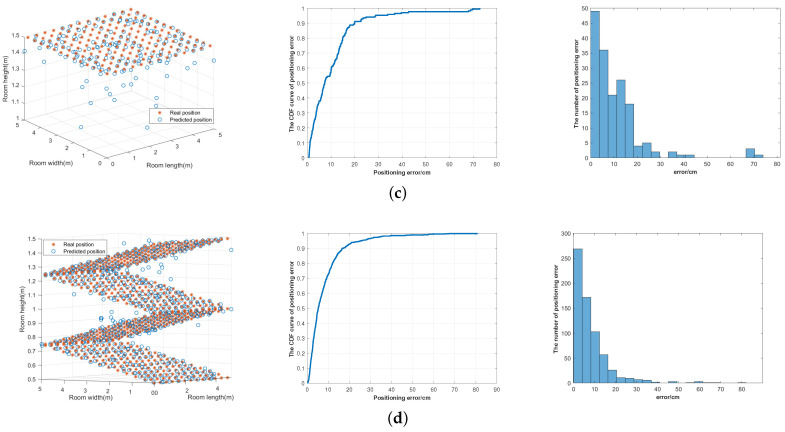
Positioning results for different test data. (**a**) 0.5 m plane. (**b**) 1.0 m plane. (**c**) 1.5 m plane. (**d**) Zigzag plane.

The above models and networks are applied to the proposed visible light positioning system. In this paper, RMSE and CDF are used to evaluate the positioning performance of the system. The specific simulation results are shown in Figure 12, showing the distribution of positioning results of four data planes and the CDF curves and histograms of corresponding positioning errors.

Figure 12a displays the result of 0.5 m plane, Figure 12b shows the result of 1 m plane, Figure 12c shows the result of 1.5 m plane, while Figure 12d shows the result of the zigzag plane. AS can be seen from Figure 12, 90% of the positioning errors in the 0.5 m plane and 1.5 m plane are below 20cm, and there are a few points with relatively large positioning errors, which are caused by the large height estimation error in the central area of the room. The positioning effect of 1m plane is better, 90% of the positioning error is less than 13 cm, and the maximum positioning error is only 25 cm. For the zigzag plane with multiple heights, 90% of the positioning errors are kept below 17%.

Table 9 shows the positioning errors corresponding to the four planes, including the maximum, minimum and average positioning errors. The maximum average positioning error is on the 0.5 m plane, which is 12.91 cm, and the minimum average positioning error is on the 1m plane, which is 5.88 cm. For the zigzag plane with multiple heights, the average positioning error is 8.22 cm. Therefore, the proposed system can satisfy centimeter-level positioning accuracy in three-dimensional space on the whole.

## 5. Conclusions

In this paper, we propose a novel indoor visible light positioning system based on point classification using artificial intelligence algorithms. When the receiver receives the RSSI data, they are first put into the height prediction model to obtain the height of the point. They are then put into the type classification model of the location point to obtain the type of the point to be positioned, and then put into the corresponding RSSI mapping model according to the type of the point to be positioned. The received power of RSSI after eliminating the multi-path effect is obtained, and the distance between the point to be positioned and each LED is then calculated according to the received power and the predicted height of the point. Finally, the position information of the point is calculated via the least square method. The system can achieve centimeter-level positioning in simulation, and meet the positioning requirements of most indoor application scenarios.

However, if we want to promote this system into real-world application, we need to consider the potential constraints and practical challenges of its implementation, such as hardware cost, model transport protocol, and indoor environmental changes.

In addition, this system also has further room for improvement. The following extensible research directions include: (1) The addition of steps and processes, such as noise reduction; (2) adoption of artificial intelligence algorithms with faster convergence speeds and enhanced capabilities; (3) further subdivision of indoor area points and the adoption of different treatment methods. 

## Figures and Tables

**Table 1:** Basic parameters for system simulation.

Parameter	Value
Emitting power of LED, $P_{t}$	4 W
Half power angle of LED, $\Phi_{1/2}$	$70^{\circ }$
Area of PD, *A*	1 cm${}^{2}$
FOV PD, $\Psi_{c}$	$70^{\circ }$
Gain of optical filter, $T_{s}\left(\Psi \right)$	1
Refractive index of the optical concentrator, *n*	1.5
Ceiling reflectance, $\rho_{ceil}$	0.8
Wall reflectance, $\rho_{wall}$	0.8
Floor reflectance, $\rho_{floor}$	0.3
Plane height interval, $d_{H}$	1 cm
Wall edge interval, $d_{wall}$	0.5 m
Corner edge interval, $d_{corner}$	1 m

**Table 2:** Performance comparison of different artificial intelligence algorithms in height estimation model training.

Artificial Intelligence Algorithm	Average Height Estimation Error (cm)
Linear Regression	32.75
Traditional SVR	12.34
GA-SVR	2.92
PSO-SVR	1.66
ANN	2.30

**Table 3:** Average height estimation error in different data planes.

Different Data Planes	Average Height Estimation Error (cm)
0.5 m	3.81
1.0 m	0.85
1.5 m	5.59
Zigzag plane	1.42

**Table 4:** Parameter settings of PSO-SVM in point classification model training.

Parameter	Value
Random seed	2000
Train data set	80%
Test data set	20%
Population number	10
Max iteration number	20
Range of c	[0.1, 100]
Range of g	[0.01, 1000]

**Table 5:** Average point classification accuracy in different data planes.

Different Data Planes	Average Point Classification Accuracy
0.5 m	99.52%
1.0 m	100%
1.5 m	99.52%
Zigzag plane	99.92%

**Table 6:** Performance comparison of different neural networks in received power mapping training.

Type of Data Set	Size of Data Set	Number of Neurons	Epoch	MSE	Average Error
Original point	34,669	64	1000	$9\times 10^{-5}$	2.61%
All data	63,125	64	1000	$2.68\times 10^{-4}$	5.1%
Edge point	23,464	128	2000	$3.35\times 10^{-5}$	3.16%
All data	63,125	128	2000	$1.8\times 10^{-4}$	4.15%
Blind point	4992	128	5000	$1.76\times 10^{-6}$	0.58%
All data	63,125	128	5000	$1.48\times 10^{-4}$	3.84%

**Table 7:** Average received power mapping error in different data planes.

Different Data Planes	Average Received Power Mapping Error
0.5 m	3.85%
1.0 m	2.67%
1.5 m	3.35%
Zigzag plane	2.65%

**Table 8:** Position error affected by received power error.

Received Power Error	Received Power (W)	Distance Calculation	Positioning Error (cm)
LOS	($4\times 10^{-5}$, $2.02\times 10^{-5}$, $8.36\times 10^{-6}$, $6.07\times 10^{-6}$)	(2.3, 2.76, 3.54, 3.86)	0.098
LOS+1% error	($4.04\times 10^{-5}$, $2.04\times 10^{-5}$, $8.44\times 10^{-6}$, $6.13\times 10^{-6}$)	(2.3, 2.77, 3.53, 3.85)	0.6
LOS+2% error	($4.08\times 10^{-5}$, $2.06\times 10^{-5}$, $8.57\times 10^{-6}$, $6.19\times 10^{-6}$)	(2.29, 2.76, 3.51, 3.84)	1.64
LOS+3% error	($4.12\times 10^{-5}$, $2.08\times 10^{-5}$, $8.61\times 10^{-6}$, $6.25\times 10^{-6}$)	(2.28, 2.75, 3.51, 3.83)	1.95
LOS+4% error	($4.16\times 10^{-5}$, $2.1\times 10^{-5}$, $8.69\times 10^{-6}$, $6.31\times 10^{-6}$)	(2.28, 2.75, 3.45, 3.82)	2.58
LOS+5% error	($4.20\times 10^{-5}$, $2.12\times 10^{-5}$, $8.78\times 10^{-6}$, $6.37\times 10^{-6}$)	(2.27, 2.74, 3.45, 3.81)	3.27

**Table 9:** Positioning errors of different test data.

Different Data Planes	Positioning Error (cm)		
	Minimum	Maximum	Average
0.5 m	0.60	82.56	12.91
1.0 m	0.25	25.88	5.88
1.5 m	0.69	72.77	10.67
Zigzag plane	0.27	80.72	8.22

## Data Availability

The data presented in this study are available on request from the corresponding author. The data are not publicly available due to secrecy restriction.

## Abbreviations

The following abbreviations are used in this manuscript:

ANN	Artificial neural network
AOA	Angle of arrival
CDF	Cumulative distribution function
FOV	Field of view
GA	Genetic algorithm
GLONASS	Global navigation satellite system
GPS	Global positioning system
LED	Light-emitting diode
LOS	Line-of-sight
NLOS	Non-line-of-sight
PD	Photo-detector
PSO	Particle swarm optimization
RFID	Ratio frequency identification
RMSE	Root mean square error
RSSI	Received signal strength indicator
SVM	Support vector machine
SVR	Support vector regression
TDOA	Time difference of arrival
TOA	Time of arrival
UWB	Ultra wide band
VLC	Visible light communication
VLP	Visible light positioning
WLAN	Wireless local area network
